# Exploring acenocoumarol and silodosin as allosteric EGFR inhibitors for the treatment of non-small cell lung cancer

**DOI:** 10.12688/f1000research.157465.1

**Published:** 2024-11-21

**Authors:** Swastika Maity, Krishnaprasad Baby, Bharath Harohalli Byregowda, Megh Pravin Vithalkar, Usha Y Nayak, K Sreedhara Ranganath Pai, Yogendra Nayak

**Affiliations:** 1 Department of Pharmacology, Manipal College of Pharmaceutical Sciences, Manipal Academy of Higher Education, Manipal, Karnataka, 576104, India; 2 Department of Pharmaceutics, Manipal College of Pharmaceutical Sciences, Manipal Academy of Higher Education, Manipal, Karnataka, 576104, India

**Keywords:** Allosteric EGFR inhibitor, NSCLC, Acenocoumarol, Silodosin, Drug repurposing

## Abstract

**Background:**

Non-small-cell lung cancer (NSCLC) is a highly morbid disease. Chemotherapy for NSCLC lacks specificity and efficacy mainly because of drug resistance. The current study aimed to explore computational tools to target allosteric epidermal growth factor receptor (EGFR) sites and screen for the top molecules *in vitro* and *in vivo* xenograft models.

**Methods:**

Molecular docking, virtual screening, and molecular dynamic studies revealed that acenocoumarol and silodosin are the top two allosteric EGFR inhibitors. They were further tested for cytotoxicity, apoptosis, cell cycle, and gene expression by qPCR, western blotting, A549 cell xenograft anti-proliferative activity, and tumor regression efficacy analysis.

**Results:**

Acenocoumarol and silodosin exhibited cytotoxicity in A549 and IMR-90 cells at concentrations below 50 and 80 μM, respectively. Acenocoumarol and silodosin induced S-phase and G2/M-phase arrest in A549 cells in the cell cycle analysis. Both drugs showed early apoptosis at their IC _50_ doses (acenocoumarol 50 μM and silodosin 25 μM). KRAS (Kirsten rat sarcoma viral oncogene homolog) and ERK2 (extracellular signal-regulated kinase 2) gene regulation in A549 cells was confirmed using qPCR. KRAS and ERK2 activities were quantified by western blot analysis. In the xenograft study, tumor size, body weight, and organ weight were significantly attenuated by the test drugs compared with the standard cisplatin. Immunoblotting and western blot results of the A549-xenograft tissue indicated downregulation of KRAS and ERK2. Furthermore, the test drugs have upregulated caspase-3 gene expression.

**Conclusion:**

The drugs acenocoumarol and silodosin downregulate KRAS and ERK2 both in cell line and in Xenograft model. KRAS and ERK2 are associated with EGFR inhibition. Hence, acenocoumarol and silodosin can be further explored for repurposing studies in human trials.

## Introduction

The surface protein receptor tyrosine kinase (TK) and epidermal growth factor receptor (EGFR) are targets for many drugs, particularly for treating lung cancer. ^1^ Mutation and alteration of EGFR in the cell lead to the development of NSCLC. Exon-19 multi-nucleotide frame deletion leads to the dimerization of four amino-acid sequences in the N-lobe of the EGFR complex, leading to single L858R and T750M or double T790M- L858R mutation condition. ^2^ A single nucleotide substitution on Exon-21 in the EGFR site leads to ATP site activation, resulting in the replacement of arginine with leucine at L858M, causing a cancerous lung condition. In NSCLC, the Cys797 mutation to serine (C797S) occurs in the EGFR moiety. ^3^ One of the major problems of EGFR-TK is autophosphorylation, leading to signal transduction pathways that activate the ATP region of the protein due to amino acid activation in the N-lobe of the EGFR complex, leading to an unstable EGFR moiety, causing cancerous growth in the cells. ^4^ Therefore, targeted therapy against EGFR overactivity may be effective for treating NSCLC. Currently, the first-generation FDA-approved EGFR therapies are gefitinib and erlotinib, which bind to the ATP site of EGFR, but they fail to inhibit autophosphorylation due to the T790 mutation occurrence. ^5^ Afatinib and dacomitinib, second-generation FDA-approved drugs, target the inactive site in EGFR and enzymatically inhibit the T790 mutation. The problem with these molecules is that they only recognize the dimeric state of EGFR and thus fail to stop EGFR overexpression in the monomeric state. ^6^ The third-generation drug, osimertinib, has a pyrimidine moiety that covalently binds to the ATP site of EGFR. This leads to the selective inhibition of T790 expression. However, with long-term use, third-generation EGFR inhibitors show C797 secondary mutations. ^7^ Tumor complexity and adaptive cell pathways for signalling in NSCLC, particularly involving the activation of various signalling pathways, such as amplification of MET/HER2, RAS-MAPK, or RAS-PI3K pathways, activate novel fusion events and histological or phenotypic transformation. ^8, 9^ Specific mutations, such as G796R, G796S, G796D, and L792H, and less prevalent mutations in exon 20 were linked with osimertinib resistance. Mutations such as C797S disrupt and weaken the covalent bond that Cys797 forms with osimertinib, preventing it from effectively targeting the mutant EGFR and thus conferring resistance to the drug. ^10^

Fourth-generation drug development involves allosteric site inhibition of EGFR. Mutant-specific allosteric inhibitors of EGFR, such as EAI001, have been found to bind to sites beyond the EGFR allosteric site. This molecule was optimized to yield EAI045, which had potency towards L858R/T790M mutations compared to wild-type EGFR. Researchers have designed and optimized allosteric EGFR inhibitors that bind to allosteric sites in the EGFR tyrosine kinase domain outside the ATP domain and have emerged as potential treatment strategies for EGFR-mutant cancers. ^11^ The main factor for choosing fourth-generation NSCLC drugs or allosteric EGFR inhibitors is that it reduces the chances of “undruggable” protein-ligand moiety formation, thus reducing the chance of adverse reactions of such drugs. ^12^ In this study, we used computational tools to identify acenocoumarol and silodosin as top-2 allosteric EGFR inhibitors. ^13^ These molecules were further evaluated for their pharmacological efficacy against NSCLC.

## Methods

### Cell culture and maintenance

A549 cells (human lung carcinoma alveolar epithelial squamous cells) and IMR-90 (fibroblasts isolated from normal fetal lungs) were procured from American-type cell culture (ATCC ^®^), and stock cells were cultured in RPMI 1640 medium (Life Technologies, Invitrogen, catalogue: 2187008), supplemented with 10% inactivated Fetal Bovine Serum (FBS, Merck Catalogue F7524), 100 IU/ml penicillin, and 100 μg/ml streptomycin (Thermo Fisher Scientific ^®^) in a humidified atmosphere of 5% CO _2_ at 37 ^o^C until confluent. The cells were dissociated with cell dissociating solution containing 0.2% trypsin (Invitrogen; catalog R-001-100), 0.02 % EDTA, and 0.05% glucose in PBS. Further, 50,000 cells/well were seeded in a 96-well plate and incubated for 24 hrs at 37 ^o^C, 5 % CO _2_ incubator (MIC-80; Microsil India Pvt. Ltd.) The cell viability is checked using a hemocytometer (Rohem Silverline Counting Chamber). The required amount of cells were further cultured in a T75 culture flask (Thermo Fisher Scientific). ^13^

### Cytotoxicity assay using MTT reagent

The top molecules from the in silico studies, danusertib, bifonazole, clopidogrel, acenocoumarol, silodosin, panobinostat, and standard doxorubicin were procured from TCI ^®^ India. The stock solution of test compounds 10 mM stocks was prepared using DMSO (dimethyl sulfoxide, TCI India). Serial two-fold dilutions were prepared from 100 μM to 3.125 μM using RPMI 1640 plain media for respective treatments. ^13^ Cytotoxicity in A549 and IMR-90 cells were carried out using MTT (3-(4,5-dimethylthiazolyl-2)-2,5-diphenyltetrazolium bromide) reagent. ^14^ The monolayer cell culture was trypsinized and the cell count was adjusted to 5 × 10 ^5^ cells/ml using respective media containing 10% FBS. To each well of the 96-well plate, 100 μl of the diluted cell suspension (50,000 cells/well) was added. After 24 h, when a partial monolayer was formed, the supernatant was flicked off, washed the monolayer once with medium and 100 μl of different test concentrations of test drugs were added onto the partial monolayer in microtiter plates. The plates were then incubated at 37 ^o^C for 24 h in 5% CO _2_ atmosphere. After incubation, the test solutions in the wells were discarded, and 100 μl of MTT (5 mg/10 ml in PBS) was added to each well. The plates were incubated for 4 h at 37 ^o^C in 5% CO _2_ atmosphere. The supernatant was removed, and 100 μl DMSO was added, and the plates were gently shaken to solubilize the formed formazan. ^15^ Absorbance was measured using a microplate reader (BMG LABTECH ^®^) at 590 nm. The percentage growth inhibition was calculated using the following formula

%Inhibition=((ODof Control–ODof sample)/ODof Control)×100.

The half maximal inhibitory concentration (IC _50_) values for cytotoxicity tests were derived from non-linear regression analysis performed using GraphPad Prism8.0 (Graph pad, San Diego, USA). Alternatively, R-program can also be used. The results were compared between groups. From the cytotoxicity results, we selected the best drugs, acenocoumarol and silodosin, for further testing.

### Apoptosis assay using propidium iodide (PI) and Annexin V-FITC staining

A549 cells (1 × 10 ^6^ in RPMI buffer), were used in this study. The plates were analyzed using a flow cytometer (FACS Calibur, BD Biosciences, San Jose, USA). After 18 h of incubation, the floating cells were replaced with the new medium. Control, standard and test samples at different concentrations were added, followed by induction of apoptosis. Cells were scraped and 1 ml of medium was pipetted to the wells. After the cells were washed twice with cold PBS, they were resuspended in a binding buffer at 1 × 10 ^6^ cells/ml. The cell suspension was portioned to 500 μl, to which 5 μl of Annexin V (Annexin V, FITC detection kit, Sigma-Aldrich ^®^) and 10 μl of propidium iodide (Sigma-Aldrich) were mixed. The plates are stored in the dark for 15 min, followed by analysis with a flow cytometer. The quadrants were created based on viable cell markers with different colors. ^16^ Gating was performed, and cell apoptosis was measured and compared in the different treatment groups.

### Cell cycle analysis

A549 cells (1 × 10 ^6^ cells/ml) were cultured and kept at 6 well plates with 2 ml of RPMI medium and incubated for 24 h. It was then subjected to treatment with the test drugs acenocoumarol (25 and 50 μM), silodosin (12.5 and 25 μM), and standard doxorubicin (25 μM) were incubated for 24 h. The cells were then harvested and centrifuged at 2000 rpm for 5 min at room temperature, and the supernatant was discarded carefully, retaining the cell pellet. The cell pellet was washed by resuspending in 2 ml of 1XPBS. The washing was repeated another time with the same conditions. The supernatant was then discarded. From the pellet, the cells were fixed by resuspending in 300 μl of Sheath fluid (BD Bioscience ^®^, catalogue no:342003), followed by the addition of 1 ml of chilled 70% ethyl alcohol drop by drop with continuous gentle shaking and another 1 ml of chilled 70% ethyl alcohol added at once. The cells were then stored at 4°C for or overnight. Post fixing, the cells were centrifuged at 2000 rpm for 5 min. The cell pellet was washed twice with 2 ml of cold 1X PBS. The cell pellet was then resuspended in 450 μl of sheath fluid containing 0.05 mg/ml PI (Sigma-Aldrich catalogue no: P4864), and 0.05 mg/ml RNaseA (Sigma-Aldrich, catalogue no: P4864) and incubated for 15 min in the dark. The percentage of cells in different stages of the cell cycle in compounds treated and untreated populations were determined using FACS Caliber (BD Biosciences, San Jose, CA). ^17^

### Western blot quantification KRAS and ERK2

A549 cells (10 × 10 ^6^ cells/2 ml) were cultured in RPMI medium, added to P35 dish, and incubated till 80% confluency. Drug treatment with acenocoumarol (25 and 50 μM), silodosin (12.5 and 25 μM), and standard doxorubicin (25 μM) were were incubated for 24 h. The protein was isolated post-harvesting by washing with PBS solution twice. The cell pellet was suspended in 300 μl of Radioimmunoprecipitation assay (RIPA) buffer (Thermo-Fisher Scientific) with 1X protease inhibitor (Sigma-Aldrich). The cells were incubated for 30 min, but every 5 min, the suspension was mixed. Then, cells were centrifuged at 10,000 rpm for 12 min and protein lysates were collected. The protein lysates were mixed with 5X loading dye and heated for 2 min at 95°C. Further, it was loaded with 10% and 15% sodium dodecyl sulphate polyacrylamide gel (SDS-PAGE) using Mini protean Tetra cell (Bio-Rad). The nitrocellulose membrane (0.2 μM) (Bio-Rad) was equilibrated in transfer buffer for 10 min. Protein transfer was done for 15 min in the Turbo Transblot (Bio-Rad) apparatus. The blot was blocked in 3% bovine serum albumin (BSA) in tris-buffered saline with Tween 20 (TBST, Thermo-Fisher Scientific, Catalogue no. A11008) for 1 h. The blot was incubated with 2° Ab (anti-Rabbit or anti-Mouse IgG-HRP (horseradish peroxidase, Thermo-Fisher Scientific) at dilution 1:10000 for 1 h. The blot was rinsed with enhanced chemiluminescence (ECL) reagent (Thermo-Fischer Scientific), for 1 min in the dark, and the images were captured between 0.5 to 5 s exposure in Chemidoc XRS and imaging system (Bio-Rad). ^17^ The protein expression levels of KRAS and ERK2 were measured, keeping glyceraldehyde-3-phosphate dehydrogenase (GAPDH) as loading control in this study and the fold regulation was calculated.

### KRAS and ERK2 gene regulation by qPCR

Treated A549 cells were scraped and washed with sterile PBS, followed by centrifugation at 12,000 rpm for 5 min at 4°C. The supernatant was discarded, and 0.4 ml of TRIzol (Invitrogen - Life Technologies ^®^) was added, mixed for 1 min, and allowed to stand for 10 min at room temperature. Chloroform (0.25 ml per 0.4 ml of TRIzol) was added, and the mixture was vortexed for 15 s. The tube was left to stand for 5 min, after which it was centrifuged at 12,000 rpm for 15 min at 4°C. The aqueous phase was carefully transferred to a new sterile microcentrifuge tube. Isopropanol (0.5 ml) was added, gently mixed for 30 s, and incubated at -20°C for 20 min. The mixture was centrifuged at 12,000 rpm for 10 min at 4°C. After removing the supernatant, the RNA pellet was washed with 0.5 ml of 70% ethanol and centrifuged at 12,000 rpm at 4°C. The supernatant was discarded, and the RNA pellet was air-dried. The pellet was resuspended in 20 μl of diethylpyrocarbonate (DEPC)-treated water, and the total RNA yield was quantified using SpectraDrop (SpectraMax i3x, Molecular Devices, USA). cDNA was synthesized from 500 ng of RNA using the PrimeScript RT reagent kit (Takara Bio) with oligo (dT) primers (Thermo-Fisher), according to the manufacturer’s instructions, in a reaction volume of 20 μl. The cDNA synthesis was performed at 50°C for 30 min. The synthesized cDNA was used for real-time polymerase chain reaction (RT-PCR) analysis. Each 20 μl PCR mixture contained 1.4 μl of cDNA, 10 μl of SYBR Green Master Mix (Thermo Fisher Scientific), and 1 μM of specific forward and reverse primers for the target genes ( Table 1). ^13^ The PCR reaction began with enzyme activation at 95°C for 2 min, followed by 39 cycles of denaturation at 95°C for 5 s, and annealing/extension at the appropriate temperature for 30 s. A secondary denaturation at 95°C for 5 s was performed, followed by a melt curve analysis from 65°C to 95°C in 5°C increments. Fold expression or regulation was calculated and compared between treated groups. ^18, 19^

**Table 1.** Primer details for KRAS and ERK2 fold regulation in A549 cells.

Sr. No.	sequence	Primer	Base pairs	Annealing temperature
**1**	TGGCACCCAGCACAATGAA	Beta actin	44	50
	CTAAGTCATAGTCCGCCTAGAAGCA			
**2**	CACGGTCATCCAGTGTTGTC	KRAS	40	50
	CACCACCCCAAAATCTCAAC			
**3**	CCGACATCTCAGGTTGGATT	ERK2	40	58
	GGTCTGTTTTCCGAGGATGA			

### Tumor regression in A549-Xenograft mouse model

The study was performed with prior approval from Institutional Animal Ethics Committee (IAEC) approval and as per the guidelines of the CPCSEA, and followed ARRIVE 2.0 checklist. ^13^ Nude mice (n=16, four groups) were used for the Xenograft induction and treatment. A549 cells at a sub-confluent level were harvested, and viable cells were counted using a haemocytometer. Post viability check, a single cell suspension of 5 x10 ^7^ cells/ml was prepared in serum-free media and mixed in matrigel at a 1:1 ratio. All the mice were subcutaneously injected with 0.2 ml of cell suspension in the region above the right flank region. ^20, 21^ Tumor size was analyzed using digital Vernier callipers. Treatment with was started when the tumor size was 100-150 mm ^3^ (2 weeks). Treatment with the test drugs and standard drugs was initiated as per the respective pre-determined doses, the positive control was intravenously administered, and test drugs were administered to animals in groups 3 and 4. Tumor length and width were measured every alternate day using Vernier calipers, and tumor volume was calculated. Once the tumor size reached the desired volume, ≈100-150 mm ^3^ (3 weeks), the tumor volume was noted and randomly sorted into four groups, each consisting of four animals. The treatment groups contained the tumor control group, to which normal saline was administered orally; the positive control group mice were treated with 4 mg/kg cisplatin (TCI, India; CAS: 15663-27-1; catalogue no: D3371), and the test sample or treatment groups III and IV received an oral dose of silodosin at 8 mg/kg and acenocoumarol at 0.2 mg/kg till 21 days. At the end of the study, the mice were euthanized. The parameters such as tumor volume, % tumor growth inhibition, % change in body weight, organ weights, mean tumor weight. ^22^ All efforts were made to reduce the suffering of the animals throughout the study phase. ^13^

### qPCR analysis of caspase-3 regulation in the Xenograft

The caspase-3 regulation in A549-xenograft tissue gene expression were analyzed and compared between the treatment groups. ^23^ The specific forward and reverse primers for the target genes are represented in Table 2.

**Table 2.** The primer sequence for gene regulation of Caspase-3 in A549 xenograft tissue.

Sr. No.	sequence	Primer	Base pairs	Annealing temperature
1	TGGCACCCAGCACAATGAA	Actin	45	60
	CTAAGTCATAGTCCGCCTAGAAGCA			
2	CAACGTCCCCTCTGAAAAA	Caspase-3	40	45
	TGGAATTGATGCGTGATGTT			

### Western blot for KARS and ERK2 in the Xenograft

Western blot analysis was performed and the Ref. 24. The respective markers were analyzed in this study.

### Histopathology of the Xenograft

The histopathology, hematoxylin and eosin (H&E) staining of tumour tissue, heart, kidney, liver, lung, and spleen were carried out. ^25^ Cell infiltration, cell rupture, and cellular confluence were analyzed to identify the effects of the treatment group compared to the control group.

## Results

### Cytotoxicity assay

**Figure 1.**
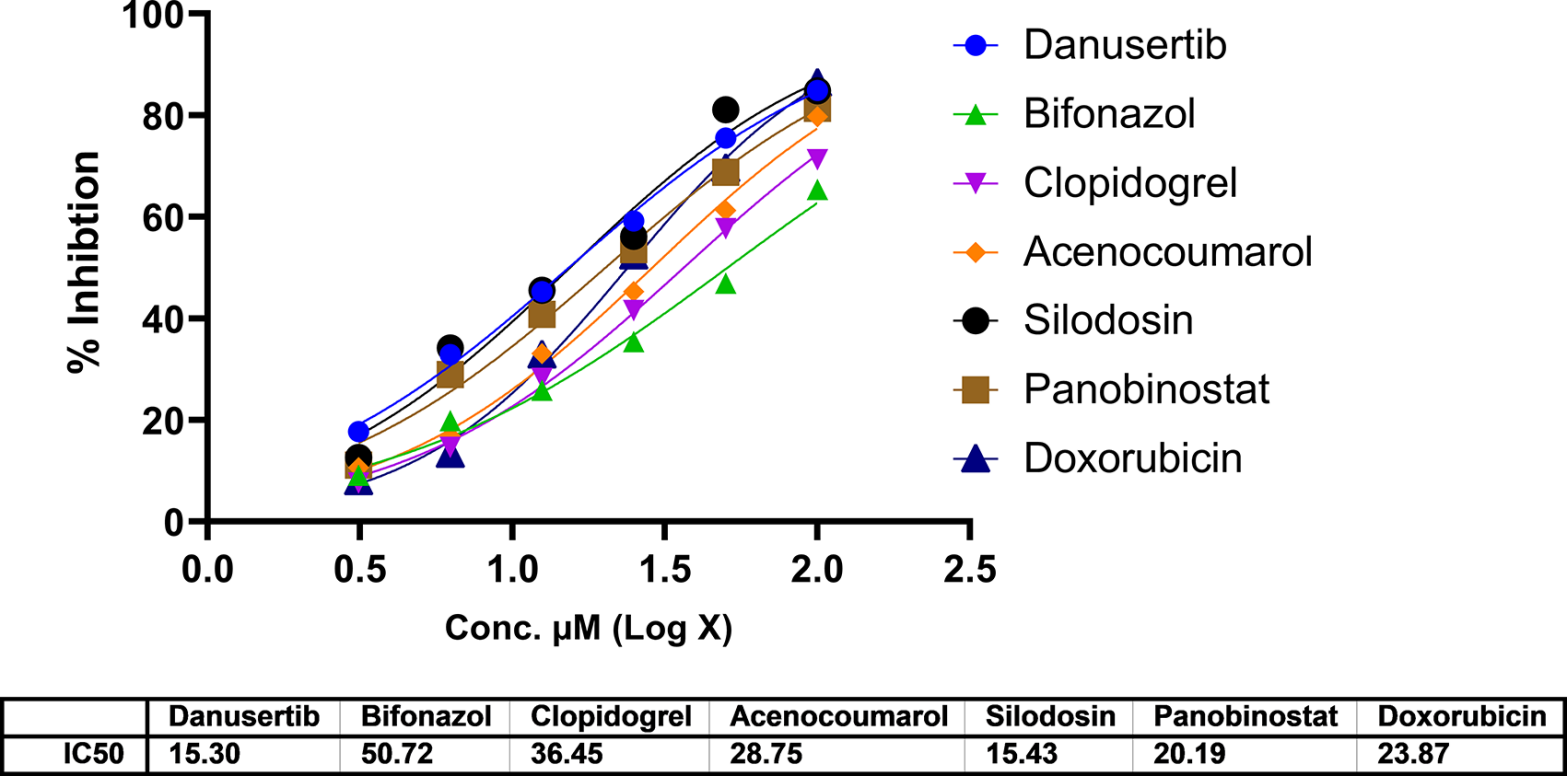
Cytotoxicity of top molecules by MTT assay using A549 cells.

**Figure 2.**
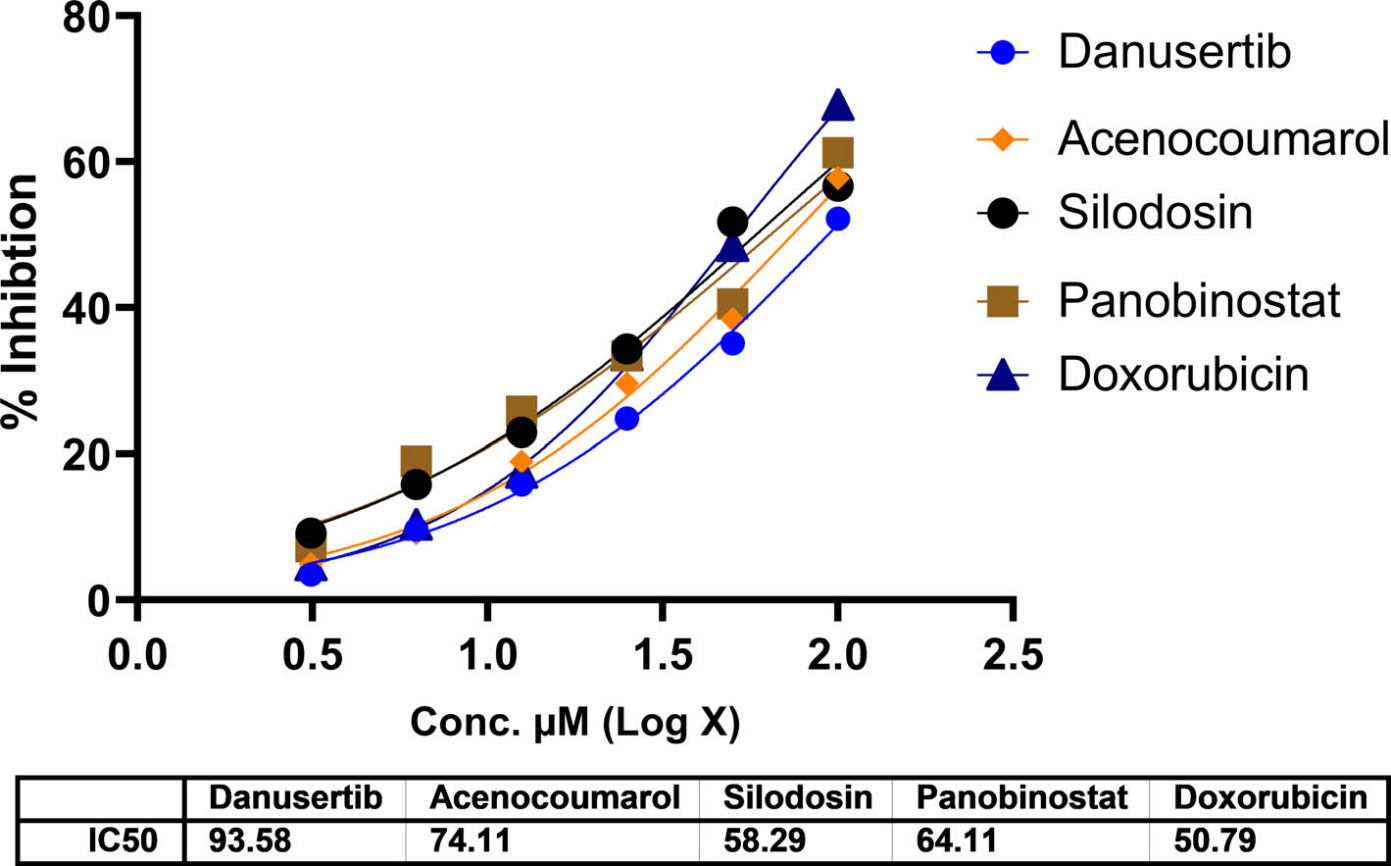
Cytotoxicity of top molecules by MTT assay using IMR-90 cells. Legend: The data presented by treatment groups is compared against control and doxorubicin. Drugs such as Bifonazol and clopidogrel did not show IC _50_, and hence, they are not represented in the figure.

In A549 cells, cytotoxicity analysis by MTT assay showed potent cell death in all treated drugs. Silodosin, danusatib, acenocoumarol, and panobinostat were more potent than the others. The IC _50_ values are presented in Figure 1. The IC _50_ in IMR-90 cells are presented in Figure 2. Acenocoumarol and silodosin were more potent than panobinostat and danusatib. Based on the IC _50_ of the drugs acting on both cell lines, acenocoumarol and silodosin were selected for further *in vitro* analysis to analyze their efficacy in treating NSCLC and their antagonistic activity as EGFR allosteric inhibitors.

### Apoptosis in A549 cells by acenocoumarol and silodosin

**Figure 3.**
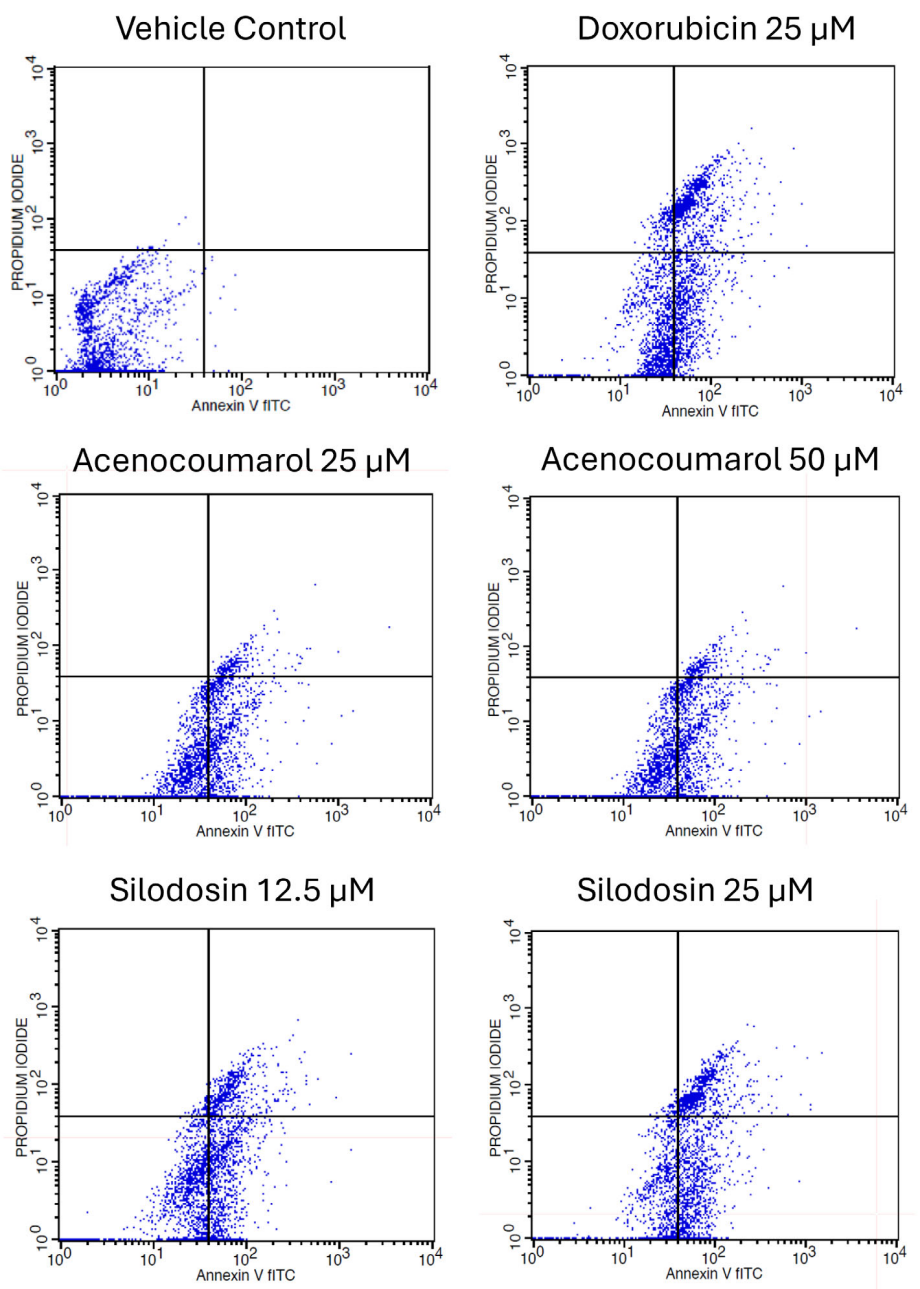
Flow cytometry plots for apoptosis detection in A549 cells. Legend: The experimental groups: vehicle control, standard control (Doxorubicin 25 μM), and Treatment groups, namely Acenocoumarol at concentrations 25 μM and 50 μM, silodosin at concentrations 12.5 and 25 μM. The viable cell population was determined at the bottom left of the quadrant of the plot. Early apoptotic cells were identified at the bottom right quadrant, and late apoptotic cells were indicated at the top right quadrant.

**Figure 4.**
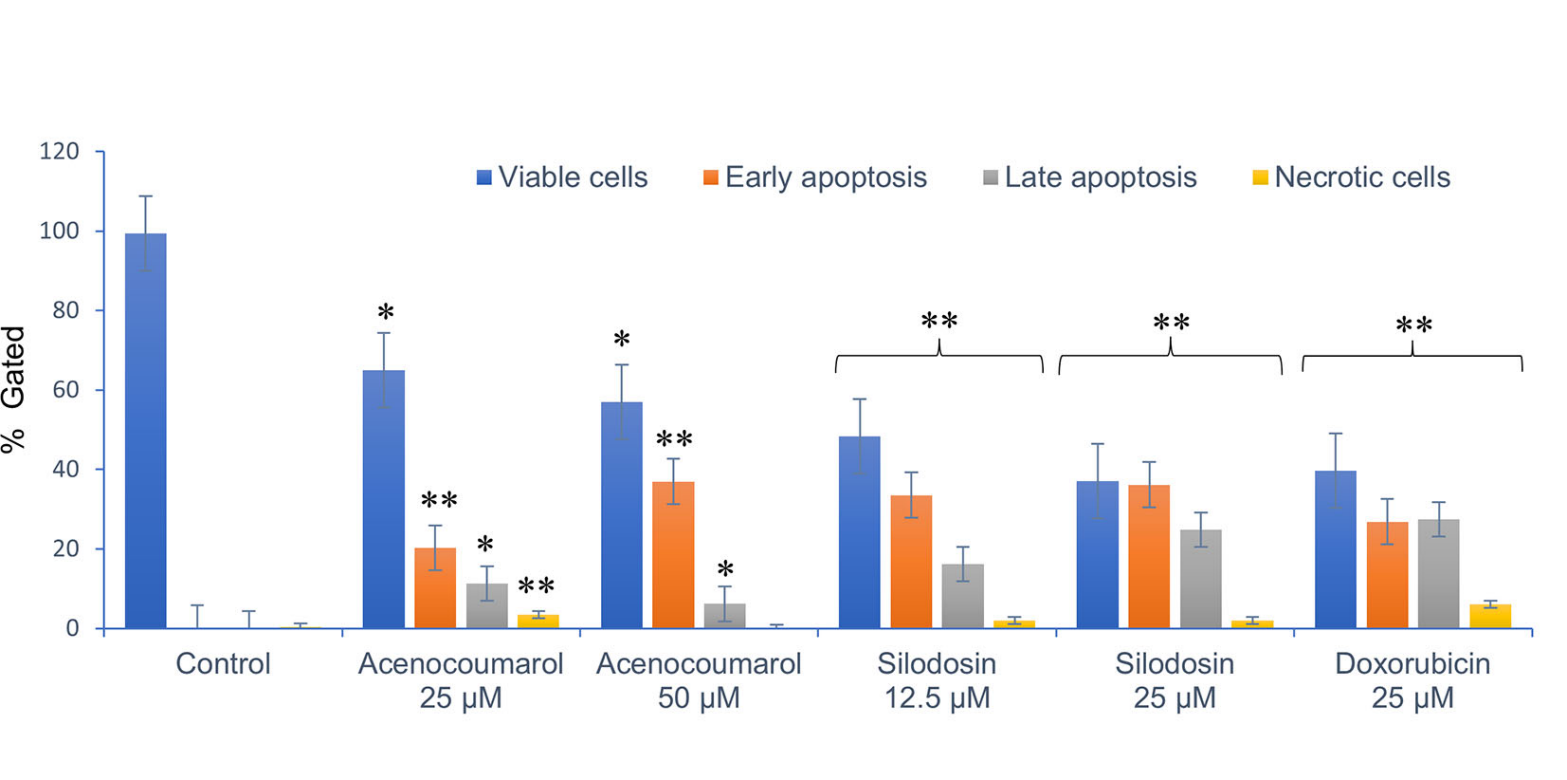
FACS analysis of apoptosis detection in A549 cells. Legend: The x-axis presents the treatment groups. Doxorubicin was the standard control. The y-axis represents the percentage of apoptosis activity. The graph shows the effect of treatment groups on checking viable cells, early apoptosis, late apoptosis and necrotic cell analysis. The data is presented by two-way ANOVA without replicas compared to doxorubicin with Mean±SEM. p<0.05, ** and p<0.5, *; compared to doxorubicin 25 μM.

Cell activity was observed in all four quadrants compared with that in the control group. The control group showed activity in the first quadrant, indicating the presence of highly viable cells ( Figure 3). This result reflects a significant decrease in cells due to the effect of treatment. The acenocoumarol treatment at 25 μM and 50 μM has induced 20.25%, 36.9% early apoptosis and 11.28%, 6.14% late apoptosis, whereas sample silodosin showed 33.49%, 36.09% early apoptosis and 16.21%, 24.84% late apoptosis at 12.5 μM and 25 μM treatment in A549 cells. Standard doxorubicin at 25 μM has shown total apoptosis of 54.24% in A549 cells ( Figure 4). The PI flow cytometry method on A549 cells in the treatment groups showed increased cell apoptosis compared to the control group. Groups such as acenocoumarol and silodosin showed earlier apoptosis when compared to doxorubicin. Silodosin at 25 μM induced more apoptosis than other treatments at different doses, as determined by Annexin V-FITC and propidium iodide (PI) staining and flow cytometry analysis.

### Effects of acenocoumarol and silodosin on A549 cell cycle

**Figure 5.**
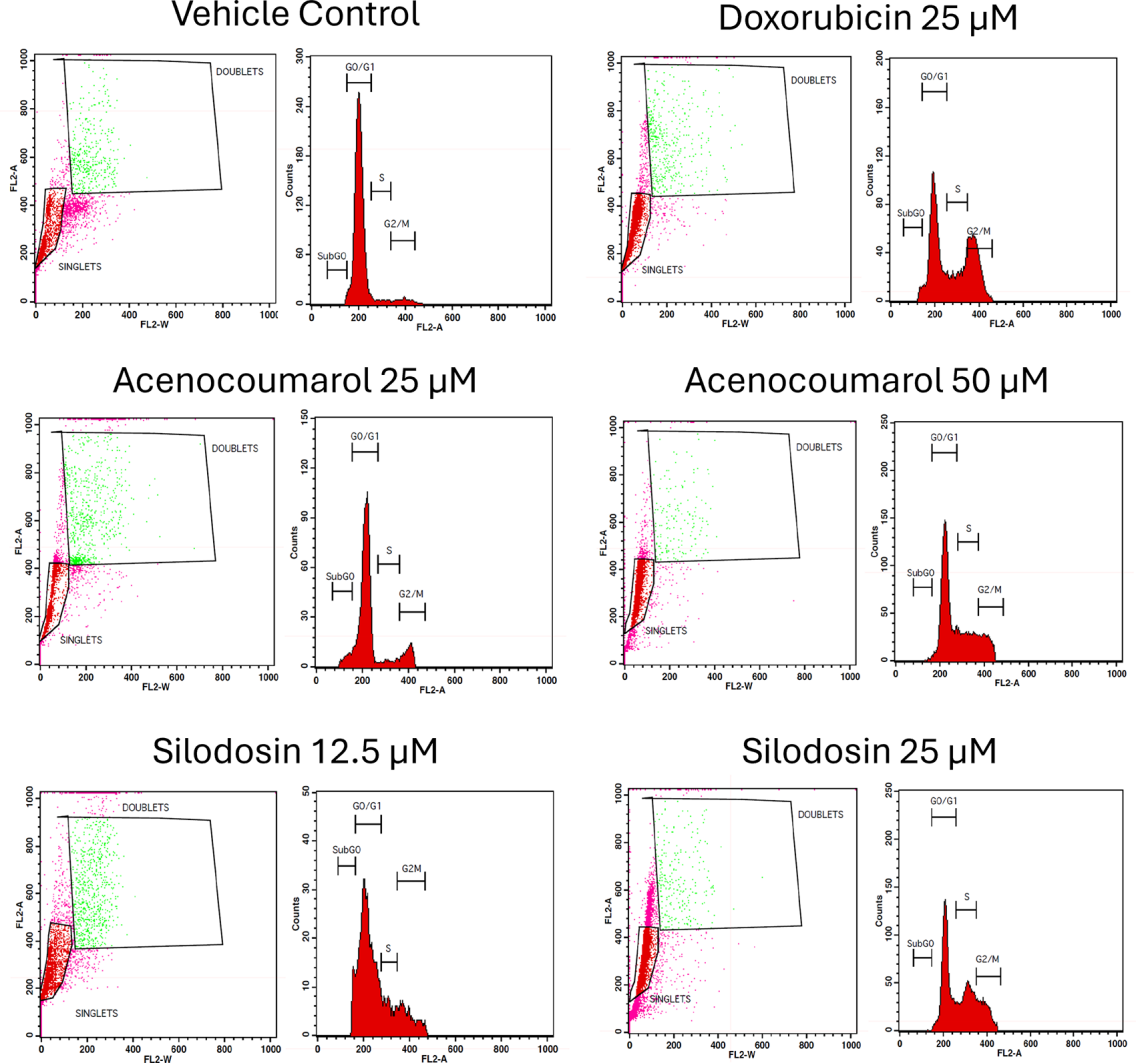
Flow cytometry plots for cell cycle analysis in A549 cells. Legend: Cell cycle activity in A549 cells with respective treatment conditions. The x-axis presents the propidium iodide-induced fluorescence; the y-axis presents the cell frequency. The stages of the cell cycle are marked as a: Go/G1, b: S, c: G2 M and d: SUB Go, respectively. Doxorubicin 25 μM, is the standard used for comparison.

**Figure 6.**
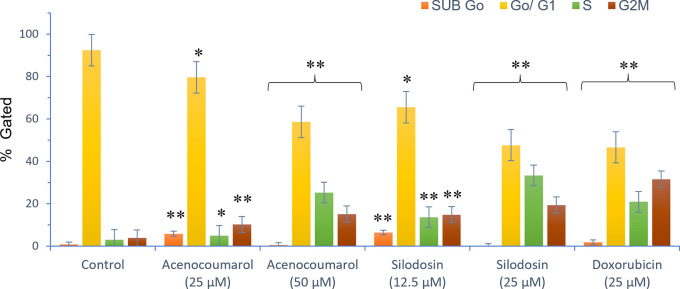
FACS analysis of Cell cycle arrest in A549 cells. Legend: Cell cycle stages are presented as SUB Go, Go/G1, S and G _2_ M phases marked with different colours. Vehicle control, Standard control (Doxorubicin 25 μM), Treatment groups namely Acenocoumarol (25 μM and 50 μM), Silodosin (12.5 and 25 μM). The data was analysed by two-way ANOVA without replicas compared to doxorubicin with Mean ± SEM. p<0.05, ** and p<0.5, *; compared to doxorubicin 25 μM.

The separation of cancer cells in the G0/1, S, and G2/M phases was demonstrated using flow cytometry analysis based on the fluorescence intensity of PI-stained cancer cells. The control group contained cells in the G0/G1 phase. The treatment of A549 cells at the concentrations of 25μM and 50μM with Sample acenocoumarol resulted in S phase and G2M phase arrest of 4.93%, 25.29%, and 10.19%, 15.15%, respectively, and Sample Silodosin has shown 13.7%, 33.37% S phase arrest, 14.82%, 19.43% G2M phase arrest at test concentrations 12.5 μM and 25 μM, respectively, in A549 cells. Standard doxorubicin at 25 μM induced G2M arrest of 31.61% and S phase arrest of 20.92%, respectively, in A549 cells. The test drugs showed significant G1 and S peaks compared with the standard control ( Figures 5 and 6). Acenocoumarol and silodosin treatment at 50 μM and 25 μM induced a significant decrease in the percentage of cells in the G1 phase, and an accumulation of cells in the S and G2/M phases of the cell cycle indicated cell cycle arrest at the S and G2M phase.

### KRAS and ERK2 enzymatic inhibition by acenocoumarol and silodosin

**Figure 7.**
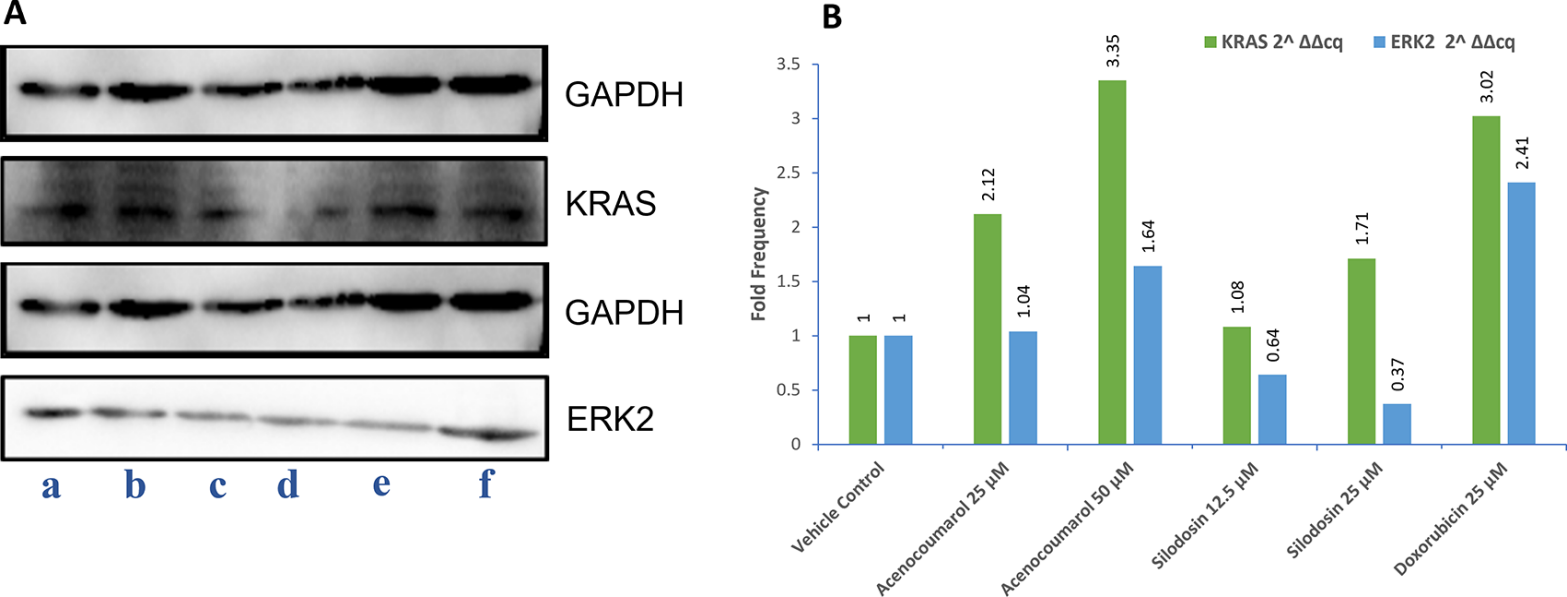
Western blot analysis of GAPDH, ERK2 and KRAS in A549 cells. Legend: A: Representation of western blot gels. The treatment groups are presented as lane a: vehicle control, b: acenocoumarol 25 μM, c: acenocoumarol 50 μM, d: silodosin 12.5 μM, e: silodosin 25 μM, f: standard control, doxorubicin 25 μM. B: Fold analysis to determine the level of expression.

The Western blot results of A549 cells treated with acenocoumarol at 25 and 50 μM concentrations suggested that KRAS and ERK2 were downregulated by 2.12-, 3.50-, 1.04-, 1.68-fold, respectively. KRAS expression was found to be downregulated in cells treated with Silodosin at 12.5 and 25 μM by up to 1.08 and 1.71 folds. ERK2 expression was upregulated when treated with Silodosin at 12.5 μM and 25 μM ( Figure 7). The standard group showed downregulation of both KRAS and ERK2 by up to 3.02 and 2.41, respectively.

### KRAS and ERK2 gene regulation by acenocoumarol and silodosin

**Figure 8.**
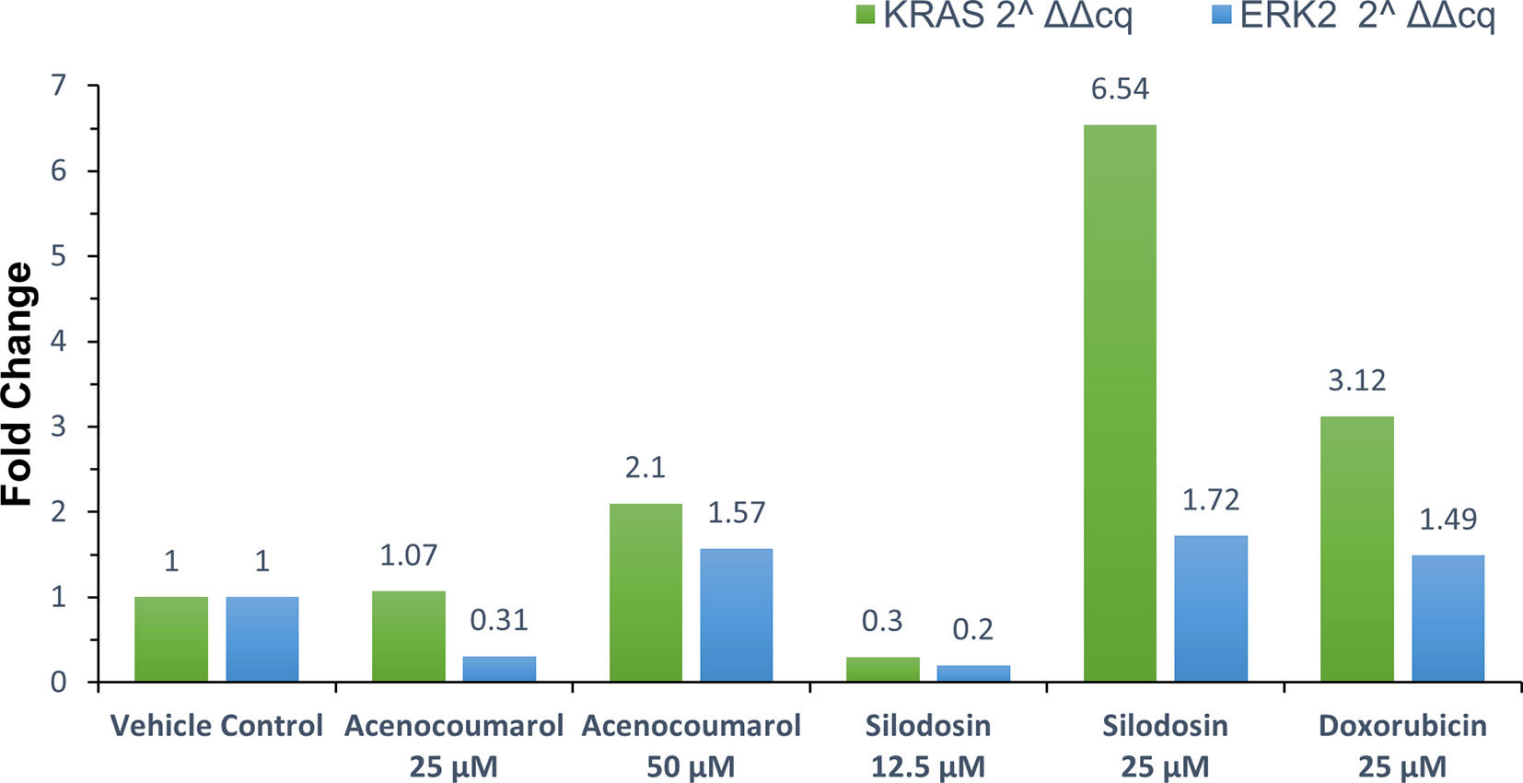
Relative expression by fold change analysis of KRAS and ERK2 gene in A549 cells. Legend: The x-axis shows the drug treatments and the y-axis shows a fold change frequency by qPCR analysis. The treatment groups are vehicle control and standard control i.e. Doxorubicin 25 μM. Treatment groups namely Acenocoumarol at concentrations 25 μM and 50 μM, and silodosin at concentrations 12.5 and 25 μM.

**Figure 9.**
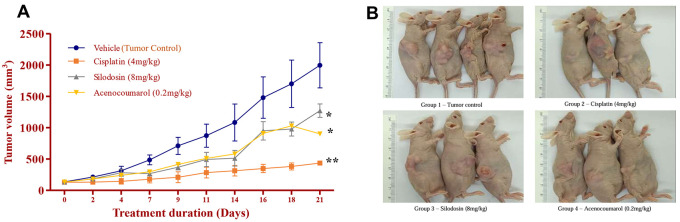
The tumour growth in A549 xenograft tumour model. Legend: A. Tumor growth profile (mm ^3^) during the treatment period of 21 days; B. Tumour induction in nude mice and its growth profile at the end of the study. In the disease control group (cisplatin 4 mg/kg), silodosin (8 mg/kg), and acenocoumarol (0.2 mg/kg), Values are represented as Mean ± SEM (n= 4). p<0.5 significant compared to the vehicle control.

qPCR or quantitative PCR analysis of the target genes KRAS and ERK2 was carried out, and fold regulation was determined. The results suggested decreased KRAS and ERK2 expression in cells treated with acenocoumarol at 50 μM compared to the control, with 2.10- and 1.57-fold expression. Acenocoumarol at 25 μM showed only a negligible effect on KRAS expression, whereas ERK2 was observed to be positively upregulated 0.31-fold ( Figure 8) compared to the control. The gene expression of both KRAS and ERK2 was upregulated in cells treated with silodosin at 12.5 μM. The expression was observed to decrease as the treatment concentration increased, with 6.54- and 1.72-fold in KRAS and ERK2 expression, respectively. The standard treatment has shown decreased expression by 3.12- and 1.49-fold in KRAS and ERK2 expression ( Figure 9).

### Tumor regression efficacy in A549 xenograft by acenocoumarol and silodosin

**Figure 10.**
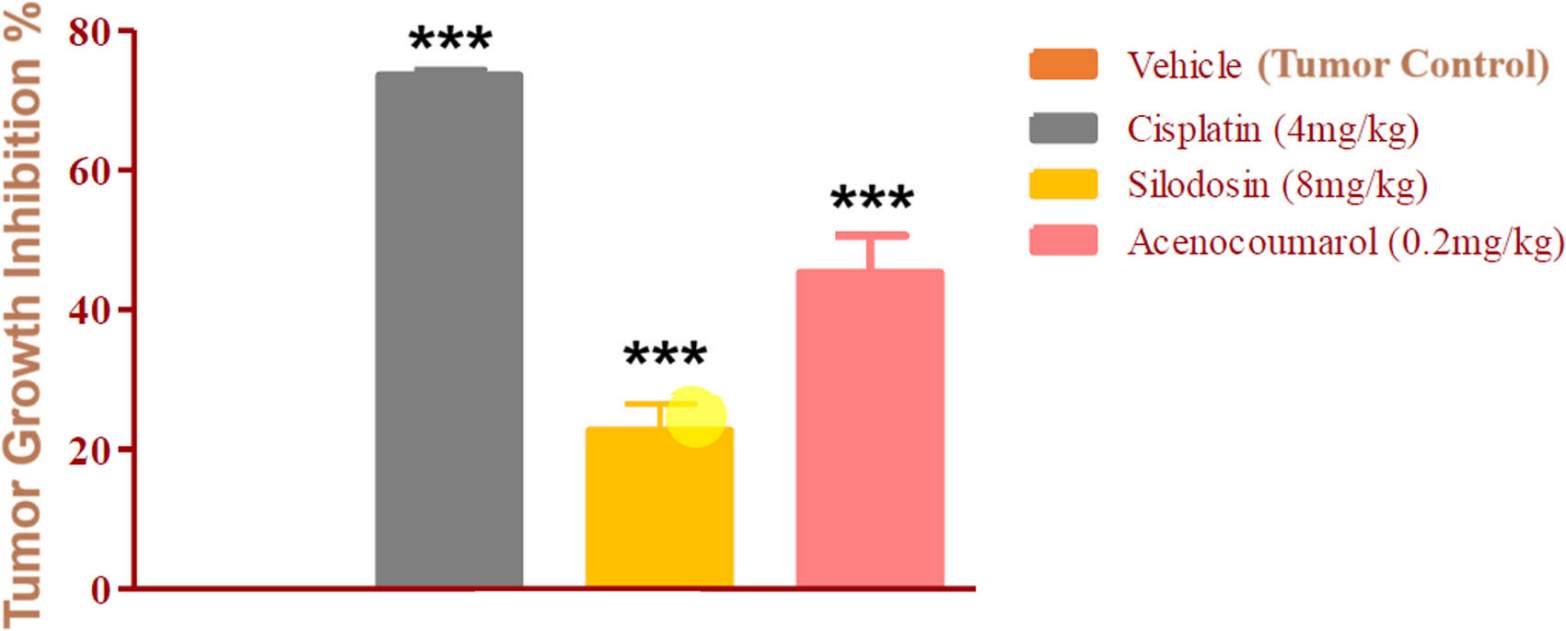
Percentage of tumour growth inhibition in the A549 xenograft tumour model. Legend: The disease control group i.e. cisplatin (4 mg/kg) showed significant tumour growth with p<0.001; the treatment groups silodosin 8 mg/kg and acenocoumarol (0.2 mg/kg) showed significant tumour growth inhibition profile against vehicle control (p<0.001). Data represented in Mean ± SEM was calculated.

**Figure 11.**
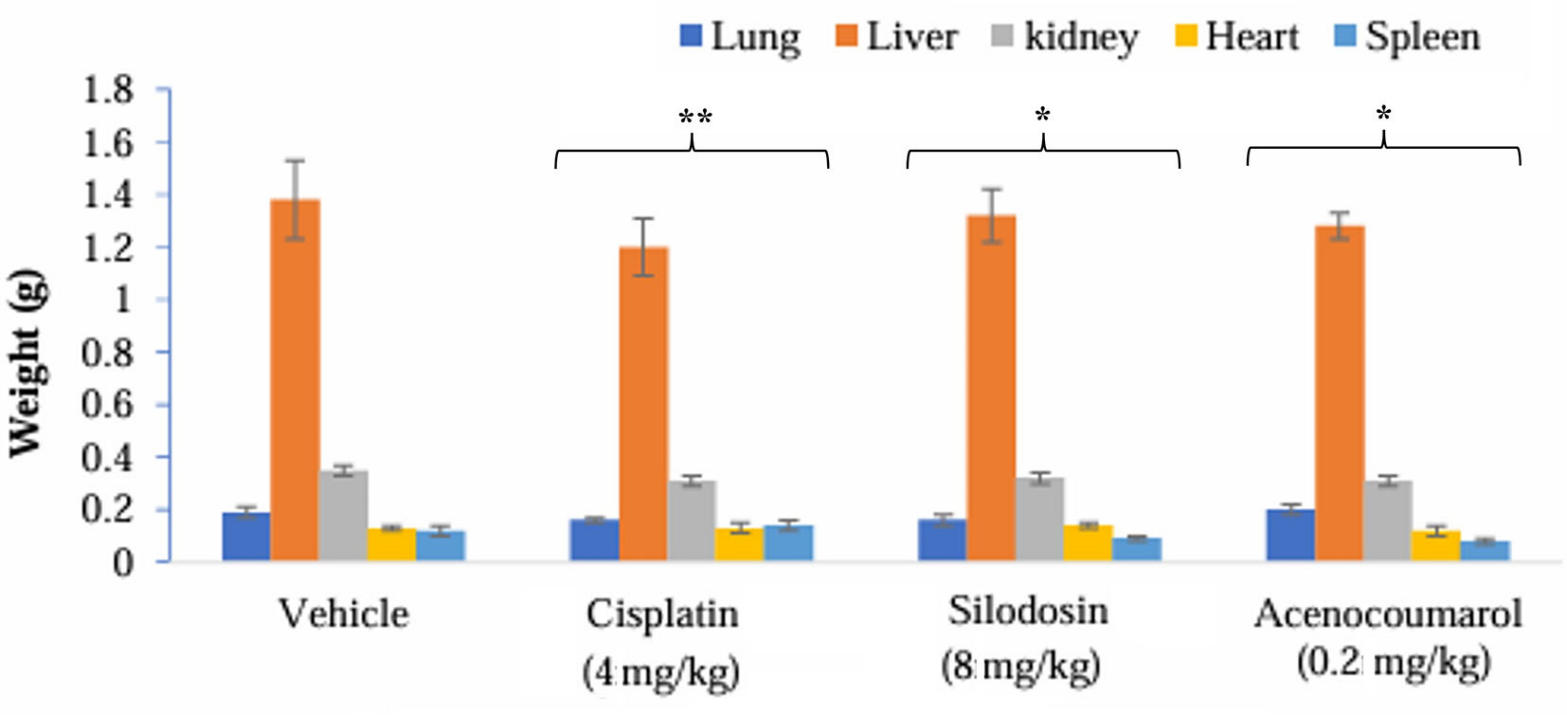
Organ weights of the mice with A549 xenograft tumour. Legend: The vehicle control is disease-induced mice, cisplatin (4 mg/kg), silodosin (8 mg/kg), and acenocoumarol (0.2 mg/kg). Values are represented as Mean ± SEM, p< 0.05 is considered significant compared to vehicle control.

The mice with A549 Xenografts treated with Cisplatin showed a tumor growth inhibition up to 73.57 ± 0.88 whereas, the test drugs showed inhibition up to 22.69 ± 3.79 and 45.3 ± 5.32% at 8 mg/kg and 0.2 mg/kg respectively (p<0.05, p<0.5) ( Figure 9). The tumor growth profile of all treatment groups was significantly reduced, indicating that the test drug showed good efficacy against NSCLC. Tumor growth inhibition was significant compared to that in control mice ( Figure 10). Other organ growth profiles suggested that the groups with treatment induction had good growth profiles compared with the disease control groups ( Figure 11).

### Caspase-3 regulation in A549 Xenograft

**Figure 12.**
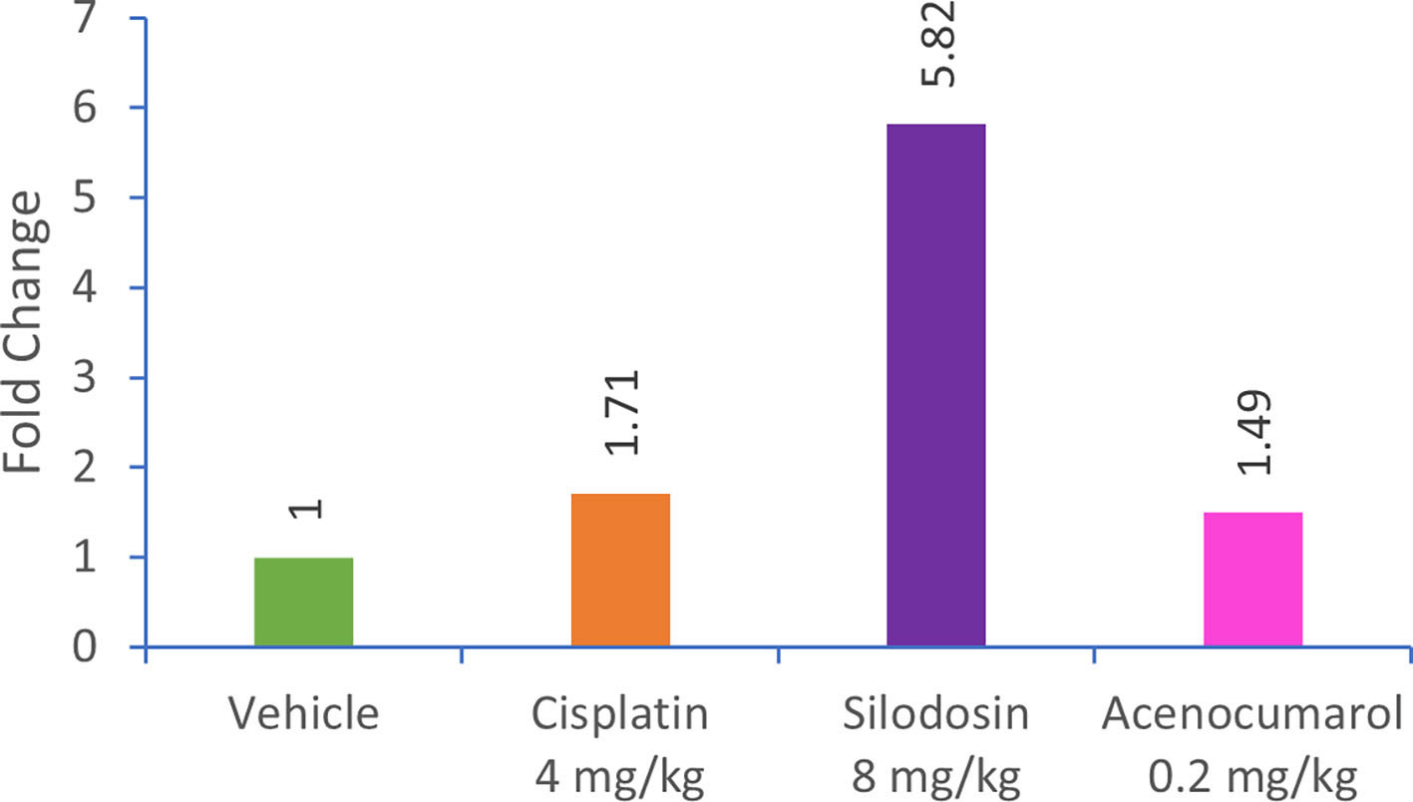
Relative expression of caspase-3 gene in xenograft tumour tissue. Legend: The treatment groups namely Silodosin (8 mg/kg), acenocoumarol (0.2 mg/kg) and the disease control group, cisplatin (4 mg/kg) were compared to vehicle control (medium).

qPCR analysis of the A549 xenograft treated with silodosin and acenocoumarol at pre-determined concentrations suggested the effective upregulation of Caspase-3 gene expression in tumor tissue samples. The tumor tissue from silodosin and acenocoumarol showed upregulation of Caspase-3 gene expression by 5.82- and 1.49-folds ( Figure 12), respectively, when compared to the control. In contrast, standard treatment with cisplatin resulted in a 1.71-fold increase in expression. Overall, the results suggest that the test sample treatment with silodosin and acenocoumarol at the respective concentrations was effective in the A549 xenograft model.

### KRAS and ERK2 enzyme inhibition by acenocoumarol and silodosin

**Figure 13.**
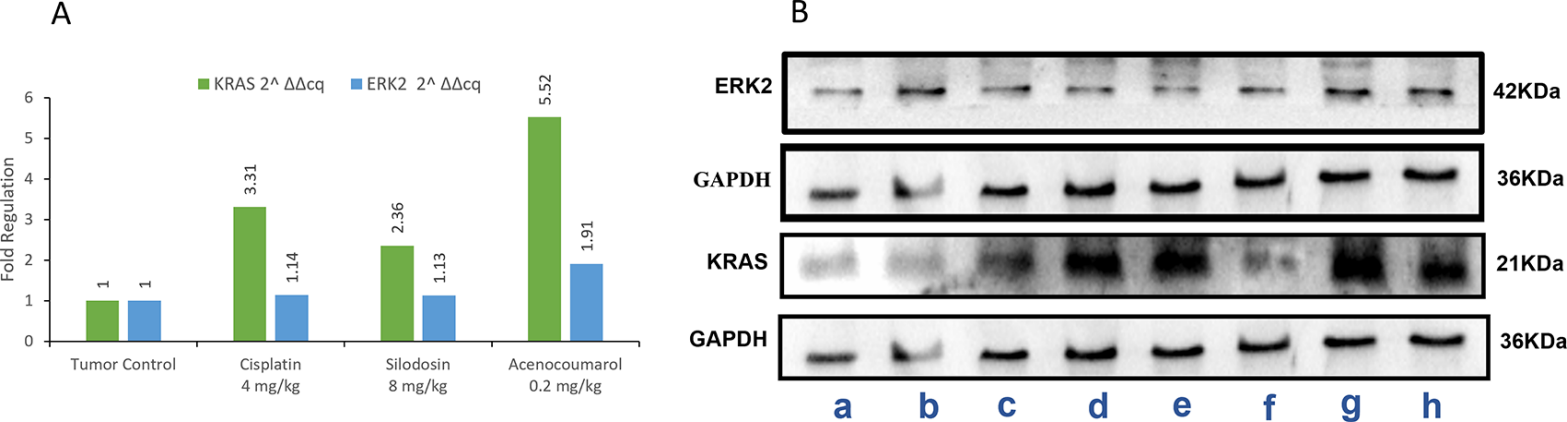
Expression analysis of GAPDH, KRAS and ERK2 in xenograft tumor model. Legend: A: The treatment groups, namely Silodosin (8 mg/kg), acenocoumarol (0.2 mg/kg) and the disease control group, cisplatin (4 mg/kg), were compared to vehicle control (medium). B: The enzymatic degradation study of KRAS, GAPDH and ERK2 marker. The groups evaluated for fold regulation were a,b: vehicle control; c,d: disease control; e,f: Silodosin (8 mg/kg) and g,h: acenocoumarol: (0.2 mg/kg).

In the blot analysis of the treatment groups, Acenocoumarol 0.2 mg/kg and silodosin (8 mg/kg) were analyzed for KRAS and ERK2 enzyme fold regulation ( Figure 13). The blots ( Figure 13B) show upregulation of KRAS, whereas ERK2 was downregulated very little, except with acenocoumarol treatment. Acenocoumarol showed better activity than the standard and silodosin treatments did.

### Histopathology analysis of Xenograft tissue

**Figure 14.**
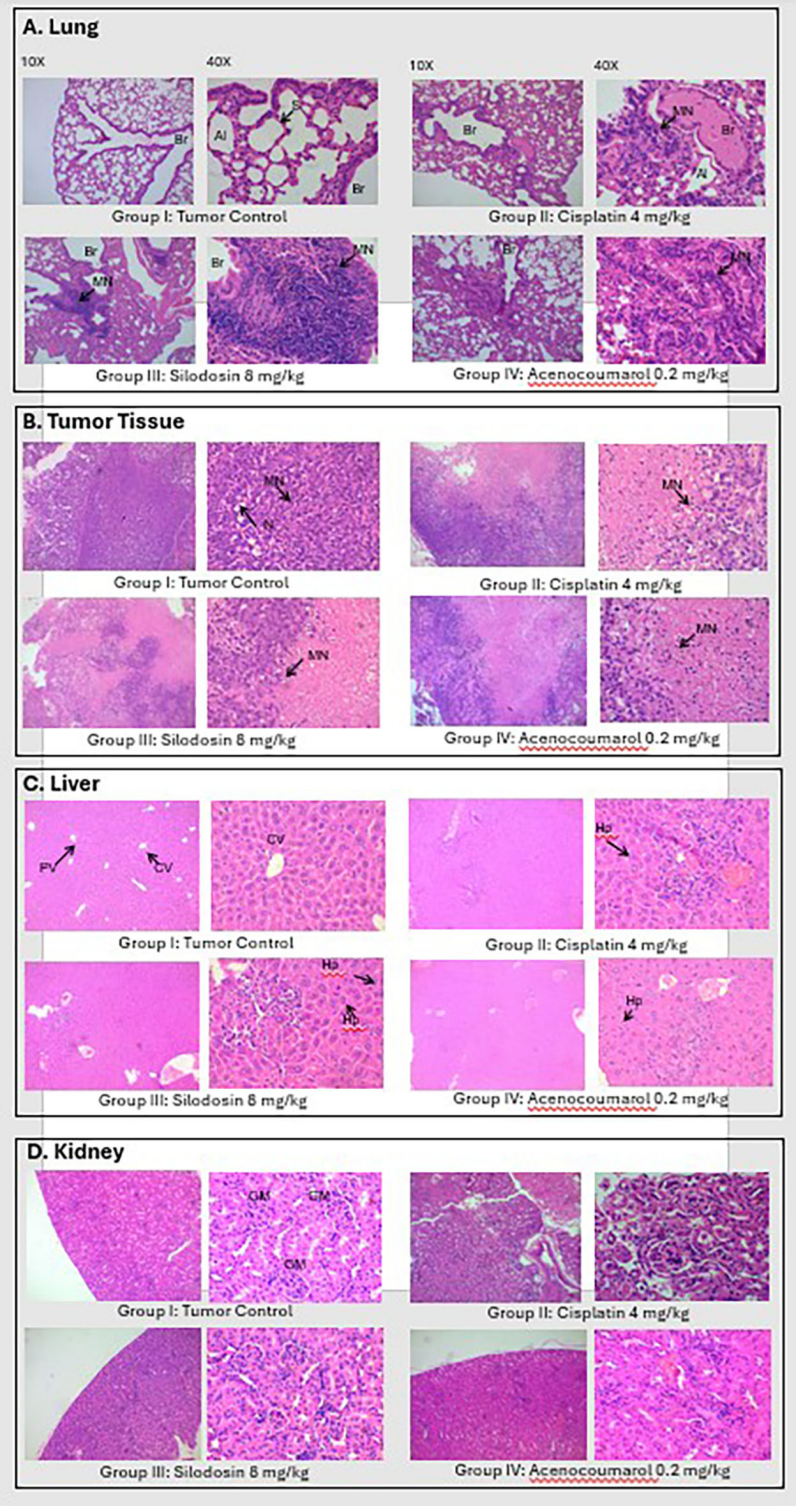
Histopathology analysis of organs obtained from xenograft model. Legend: A: lungs, B: Tumor, C: Liver and D: Kidney.

Tumor analysis of the histopathology test ( Figure 14) showed that A/group I: Normal architecture was observed. B/group II: Necrosis with moderate mononuclear cell infiltration. C/group III: mild necrosis with mild mononuclear cell infiltration. D/group IV: moderate mononuclear cells. Silodosin and acenocoumarol showed good anti-NSCLC activity compared with the disease control group. The arrows in Figure 14 show cell infiltration/inflammation. The treatment groups showed less cell infiltration than the vehicle-disease control group, indicating suppression of cancerous activity.

## Discussion

EGFR is the major oncogene driver in NSCLC, and EGFR-targeted therapy has the potential to deliver greater suppression of aggressive NSCLC compared to cytotoxicity-based pharmacotherapy. ^26^ Drugs that target the allosteric site of EGFR-TK, disrupt the cell-signalling cascade and alter cellular function. ^27^ The allosteric site has attracted significant interest from researchers and is now regarded as a highly promising target for developing anti-NSCLC drugs. Therefore, this study highlights the significance of inhibiting the EGFR allosteric site, which intersects with the TK receptor-ligand interaction. ^28^ It has also been stated that anaplastic lymphoma kinase (ALK), reactive oxygen species (ROS1), kinase-renin-angiotensin system (KRAS), protein kinase B (PKB), or (AKT) are other validated tissue biomarkers in NSCLC due to its downregulation of EGFR kinase in cell proliferation. ^29^ Targeting the allosteric site stabilizes the protein complex, influencing the binding efficiency of the primary ligand. This inactivates the protein and reduces the responsiveness to ligands or results in a neutral effect during ligand interactions. Consequently, allosteric sites have the ability to disrupt or fully halt the signal transduction process. Moreover, the site remained stable throughout the interaction. As a result, allosteric site targeting can effectively suppress cellular proliferation, particularly in malignant conditions, with allosteric inhibitors showing high selectivity. ^12^

Acenocoumarol is an oral anticoagulant primarily used for the prevention and treatment of thromboembolic disorders. It inhibits the synthesis of vitamin K-dependent clotting factors (II, VII, IX, and X) in the liver, which are essential for blood clot formation. This action helps prevent the formation of new blood clots and the growth of existing clots. ^30^ Silodosin is an alpha-1 adrenergic receptor antagonist primarily used for the treatment of benign prostatic hyperplasia (BPH), a condition characterized by an enlarged prostate gland that can cause urinary symptoms in men. ^31^ Acenocoumarol is a coumarin derivative with an active chiral core that is present in the enantiomeric form. ^32^ Acenocoumarol is absorbed by the gastrointestinal tract and undergoes first-pass metabolism, which results in peak plasma levels after dosing. In addition, if not administered in lower doses, it may lead to thromboembolic events. ^33^ A study indicated that acenocoumarol can upregulate extracellular signal-regulated kinase 2 (ERK1/2), leading to the activation of phosphorylation in the TK pathway. Acenocoumarol also affects the PI3K/AkT, MAPK, and RAS pathways by controlling the TK moiety. ^34^

Enzymes ERK2 and KRAS significantly inhibited enzymatic activity upon administration of silodosin. Hence, silodosin inhibits cell proliferation in NSCLC by inhibiting the signal transduction cascade, MAPKs, ERK, and k-RAS, which can stop aggressive growth, leading to potent anti-NSCLC activity. An earlier report showed that silodosin was able to produce pro-apoptotic activity in bladder cancer cells by enhancing the cytotoxic activity of cisplatin via ELK1 inactivation. ^35^ Silodosin is produced by α-androgenic blockers. Silodosin is the treatment of choice for lower urinary tract symptoms (LUTS) and benign prostatic hyperplasia (BPH). ^36^ Signal transduction in the TK complex via phosphorylation at certain sites in response to MAPKs and ERK. ELK-1 activation has been reported to demonstrate pro-apoptotic activity within the cytoplasm and pro-differentiation activity inside the nucleus of cancer cells, which lowers aggressive cell proliferation. ^37^ The degradation products of silodosin also exhibited anticancer activity. ^38^ Hence, silodosin could be repurposed as an anti-NSCLC drug.

Allosteric inhibition of EGFR can prevent the autophosphorylation of tyrosine residues, blocking the recruitment of Grb2 (Growth factor receptor-bound protein 2) and Son of Sevenless (SOS), which are essential for KRAS activation. ^39^ ERK2 is the downstream regulator of KRAS in the RAS-RAF-MEK-ERK pathway; hence, activation depends on upstream signals, such as EGFR. ^40^ Hence, allosteric EGFR affects the activation of KRAS, which cascades through RAF and MEK to influence ERK2 phosphorylation. In this study, when enzymes ERK2 and KRAS were examined for their activity following the administration of silodosin, they demonstrated upregulated activity by exhibiting considerable enzymatic inhibition. We can conclude that silodosin and acenocomerol were able to reduce cell proliferation in NSCLC by suppressing the signal transduction cascade, MAPKs, ERK, and KRAS. Silodosin was able to promote pro-apoptotic activity in cancer cells and inhibit aggressive cancer growth, leading to significant anti-NSCLC activity that could be attributed to its allosteric EGFR inhibition.

## Conclusion

The *in silico* study inferred that acenocoumarol and silodosin have an affinity for EGFR allosteric sites. Acenocoumarol and silodosin were subjected to a series of *in vitro* and A549 xenograft analyses to evaluate their potencies. Both drugs showed significant effects on A549 cells and were able to control the overexpression of ERK2 and KRAS. These drugs can be further explored in clinical trials to determine the efficacy of these drugs as fourth-generation anti-NSCLC agents.

## Ethics considerations

The study was approved by the Institute’s Ethical Committee as per IAEC guidelines, and the Arrive Guidelines 2.0 was archived. The ethics approval no. for the study was IAEC-SLS-2022-071 (Date: 25 ^th^ October 2022). All studies and animal handling were performed strictly according to the IAEC guidelines, and the ARRIVE 2.0 Checklist was archived. ^13^

## Author contributions

Swastika Maity: Conceptualization, Data curation, formal analysis, Investigation, Methodology, Project administration, writing the original draft, and editing.

Krishnaprasad Baby: Methodology, Formal analysis, Validation

Bharath Harohalli Byregowda: Methodology, Formal analysis, writing- review and editing

Megh Pravin Vithalkar: Methodology, Formal analysis, Visualization

Usha Y Nayak: Supervision, Validation, Resources

K Sreedhara Ranganath Pai: Supervision, Validation, Conceptualization.

Yogendra Nayak: Conceptualization, formal analysis, methodology, data curation, supervision, funding acquisition, writing, review, and editing.

## Data Availability

Underlying data archived at Figshare https://doi.org/10.6084/m9.figshare.24587592.v5. ^13^ This project contains the following underlying data: ARRIVE-2.0_checklist.pdf Supplementary_file-1.docx (Data generated by in silico studies) Supplementary_file-2.docx (Data generated by in vitro and in vivo studies) Methods in Detail 1.docx (Detailed materials and methods for in vitro and in vivo studies) Results.xlsx (Contains the in vitro and in vivo data) Images- SDS.pptx (Contains the original images of western blots)
