# Supplementary material for: Exploring acenocoumarol and silodosin in non-small cell lung cancer: Insights into EGFR-linked signaling mechanisms
Source: F1000Res. 2025 Sep 23;13:1398. Originally published 2024 Nov 21. [Version 2] doi: 10.12688/f1000research.157465.2 (PMC12728339; doi:10.12688/f1000research.157465.2)
Supplement: Peer review history

# Reviewer report: Reviewer response for version 2

**DOI:** 10.5256/f1000research.188258.r434404

**Published:** 2025-12-29

**Authors:** Abhijeet Rajendra Joshi

**Affiliations:** 1 Birla Institute of Technology and Sciences Pilani, Hyderabad, Hyderabad, India

- Citation #17 in the Western blot methods is not correct. The citation is for flow cytometry.
- Citation #25 for the histopathology is not correct.
- For the cytotoxicity assay in the results, the authors mention “Acenocoumarol and silodosin were more potent than panobinostat and danusatib.”. Without a proper statistical analysis, the statement is inappropriate.
- Fig 7: The blots for both GAPDH are same. They blot of GAPDH should be different for different proteins. Same for Fig 13. Also, there is no statistical test applied for the quantification. It is not appropriate to demonstrate the changes in the protein expression without proper statistics.
- Same for Fig 8. No statistical tests applied.
- Fig 10: There is some dot on the graph. Please rectify.
- Fig 11: The * values are not clear. Which organ is being compared here? Which difference is being shown by * and ** is not clear.
- Fig 14: What are the arrows pointing is not clear in the captions.

Is the work clearly and accurately presented and does it cite the current literature?

Partly

If applicable, is the statistical analysis and its interpretation appropriate?

Partly

Are all the source data underlying the results available to ensure full reproducibility?

Yes

Is the study design appropriate and is the work technically sound?

Yes

Are the conclusions drawn adequately supported by the results?

Partly

Are sufficient details of methods and analysis provided to allow replication by others?

Yes

Reviewer Expertise:

Neuroscience, Immunology

I confirm that I have read this submission and believe that I have an appropriate level of expertise to confirm that it is of an acceptable scientific standard, however I have significant reservations, as outlined above.

# Reviewer report: Reviewer response for version 2

**DOI:** 10.5256/f1000research.188258.r434405

**Published:** 2025-12-23

**Authors:** Navjot Kanwar

**Affiliations:** 1 Maharaja Ranjit Singh Technical University, Bathinda, Punjab, India

1. The authors should update the abstract to exclude the repetitive references to EGF-linked signalling pathways, the same mechanism principle appears several times and reducing all of these into a single brief description improves clarity and readability.
2. In the introduction, the authors must explain the limitations of existing EGFR inhibitors.
3. The author had already discussed non-allosteric EGFR inhibitors (such as EAI001, EAI045, JNJ-09-063, or other fourth-generation inhibitors). The authors make scientific claims but don’t provide enough references to support these statements.

 The author must explain

- How previous inhibitors work,
- Type of mutation they target,
- Their potency
- Mechanism of action
- Limitations

Must include relevant citations.

 4. Although the authors defend the use of acenocoumarol and silodosin, this justification would be strengthened if more detailed docking metrics, such as docking scores, MM-GBSA energies, and comparative ranking among all screen compounds, were provided.

 5. The rationale for selecting the specific *in vitro* concentrations (e.g., 25 and 50 μM for acenocoumarol, 12.5 and 25 μM for silodosin) and the *in vivo* doses (0.2 mg/kg acenocoumarol, 8mg/kg silodosin) could be explained more clearly. The author should indicate whether these exposures are within, above, or far beyond clinically achievable plasma concentration, and briefly explain how this impacts translational feasibility.

 6. Since cisplatin was used as a general antitumour benchmark rather than an EGFR-specific control, the authors could add a short paragraph explaining how the relative magnitudes of tumour inhibition (cisplatin vs test drug) should be interpreted.

 7.The manuscript currently lacks direct measurement of key signalling markers such as phosphor-EGFR, phosphor-ERK1/2 and active GTP-bound RAS. The authors must provide this in the limitations section and indicate that future work will incorporate phospho-protein assays (e.g., p-EGFR, p-ERK1/2), RAS GDP pulldown experiments or broader phosphor-proteomics to more definitively characterise pathway modulation.

 8. There is a clear conflict between the qPCR or (mRNA) data and the corresponding protein levels for KRAS and ERK2. The authors should briefly discuss possible biological reasons for this mismatch and indicate that time-course studies for proteomic analysis would be required to resolve these differences.

 9. The authors should briefly discuss potential roles of post-translational regulation, protein turnover, and feedback from upstream or parallel pathways (e.g., PI3K/AKT/MET/HER2) that could account for partial uncoupling of transcript and protein levels at a single time point.

 10.The flow cytometry procedures are explained appropriately; however, the authors must include how they selected specific populations (e.g., FSC/SSC, singlet gating and final analysis gates) and adding those gating strategy images would enhance clarity and ensure reproducibility of apoptosis and cell cycle results.

 11.The small sample size n = 4 per group, statistical power and make it difficult to generate strong outcomes, the authors should acknowledge this limitation and indicate that the *in* *vivo* findings should be interpreted as preliminary and hypothesis‑generating rather than definitive efficacy data.

 12. The authors must include key limitations of their study, including pharmacokinetic concerns and the absence of direct biochemical evidence confirming EGFR inhibition, which helps to maintain a balanced and scientifically updated interpretation of their outcomes.

 13. The xenograft data indicate meaningful tumour suppression; however, the authors must include clinically relevant EGFR inhibitors such as a positive control would significantly improve the evaluation and contextual interpretation of treatment efficacy.

 14. The histopathological findings support the anti-cancer activity of the compounds; however, the authors must provide higher resolution micrographs with clear scale bars and magnification to ensure adequate visualisation of tissue morphology.

 15. For the Western blots figures, the author should clarify in the figure description how bad intensities were normalised (e.g., to GAPDH or another loading control) and confirmed that the blots represent independent biological replicates rather than reprobing of the same membrane, this information is necessary to ensure proper interpretation and reproducibility of the result.

 16. For multi endpoint figures (apoptosis cell cycle xenograft tumour volumes) the statistical comparison would be clearer if exact P value were included.

 17. The authors must provide more detail in quantitative graphs such as access labelling to improve interpretation and presentation quality. For example,

- Percentage of apoptotic cells
- Early vs late apoptosis
- Treatment groups
- Concentration units
- 18. The authors state that one-way ANOVA with Tukey’s post hoc tests were used and the data are presented as mean ± SEM, it would be helpful to specify for each figure whether n refers to biological replicate (independent experiments or animals) and to clarify how many technical replicates were included and this information is present in parts of methods but should be included in figure description.
- 19.The authors must include software versions, grid definitions, scoring functions, ligand preparation steps, selected force fields, solvation models, equilibrium protocols, and simulation length in the computational method for docking and MD stimulation.

Is the work clearly and accurately presented and does it cite the current literature?

Yes

If applicable, is the statistical analysis and its interpretation appropriate?

Partly

Are all the source data underlying the results available to ensure full reproducibility?

No source data required

Is the study design appropriate and is the work technically sound?

Yes

Are the conclusions drawn adequately supported by the results?

Yes

Are sufficient details of methods and analysis provided to allow replication by others?

Yes

Reviewer Expertise:

Drug discovery and formulation development

I confirm that I have read this submission and believe that I have an appropriate level of expertise to confirm that it is of an acceptable scientific standard, however I have significant reservations, as outlined above.

# Reviewer report: Reviewer response for version 2

**DOI:** 10.5256/f1000research.188258.r423555

**Published:** 2025-11-11

**Authors:** Jigna Samir Shah

**Affiliations:** 1 Nirma University, Ahmedabad, Gujarat, India

This revised manuscript presents an integrative exploration into the repositioning of acenocoumarol and silodosin for the treatment of non-small cell lung cancer (NSCLC), with a particular focus on their modulation of EGFR-associated signaling cascades. The study draws strength from its translational workflow, blending computational docking, cellular assays, and in vivo xenograft investigations, which adds both depth and multidimensionality to its findings.

 The authors have responded judiciously to previous reviewer comments, demonstrating considerable effort in refining the manuscript structure, improving scientific phrasing, and moderating interpretative assertions. The most commendable revision is the recalibration of the central claim: the manuscript now appropriately frames these compounds as *putative binders at EGFR-associated regions* rather than confirmed allosteric inhibitors. This responsible repositioning respects the bounds of evidence while preserving scientific enthusiasm.

 The experimental design is logical and builds progressively. The **in silico molecular docking data** suggest favorable binding affinities for acenocoumarol and silodosin at sites adjacent to the canonical ATP-binding pocket of EGFR. These insights are augmented by **in vitro studies**, which demonstrate downregulation of KRAS and ERK2 in A549 cells, two key downstream regulators in the EGFR-RAS-RAF-MEK-ERK axis. Furthermore, the **in vivo xenograft findings** in mice corroborate these effects, lending preliminary yet meaningful validation to the biological relevance of the compounds under investigation.

 In the revised version, the **conclusion has been carefully moderated**. The revised phrasing avoids prematurely positioning these agents as clinical candidates and instead articulates the need for further mechanistic and translational research. Additionally, the **keywords have been updated** to more accurately reflect the refined scientific scope of the study.

 The **discussion section has been significantly improved**, now offering a thoughtful comparison with known allosteric EGFR inhibitors such as EAI045 and JBJ-09-063. The authors clearly articulate that while experimental validation of EGFR binding is not yet available for the test compounds, the predicted interaction profile opens up possibilities for future analog development or combination therapies.

 A brief but important discussion on the pharmacokinetics and safety limitations of acenocoumarol and silodosin has been thoughtfully included. The authors responsibly note the bleeding risks, metabolic concerns, and drug interaction profiles of these agents, offering a realistic assessment of their translational feasibility.

 From a scholarly standpoint, the manuscript is now more cohesive, restrained in tone, and aligned with the principles of hypothesis-driven science. While experimental limitations exist, as is common in early-stage repositioning research, the work sets a solid foundation for further inquiry and is likely to benefit researchers engaged in EGFR-targeted therapy development.

Is the work clearly and accurately presented and does it cite the current literature?

Yes

If applicable, is the statistical analysis and its interpretation appropriate?

Yes

Are all the source data underlying the results available to ensure full reproducibility?

Yes

Is the study design appropriate and is the work technically sound?

Yes

Are the conclusions drawn adequately supported by the results?

Yes

Are sufficient details of methods and analysis provided to allow replication by others?

Yes

Reviewer Expertise:

Oncology, neurodegenerative disorders, metabolic disorders.

I confirm that I have read this submission and believe that I have an appropriate level of expertise to confirm that it is of an acceptable scientific standard.

# Reviewer report: Reviewer response for version 1

**DOI:** 10.5256/f1000research.172916.r347134

**Published:** 2025-01-03

**Authors:** Chandan Shivamallu

**Affiliations:** 1 Department of Biotechnology and Bioinformatics, JSS Academy of Higher Education and Research, Mysuru, India

This manuscript presents an innovative approach to repurpose acenocoumarol and silodosin as allosteric inhibitors targeting EGFR for non-small-cell lung cancer (NSCLC). The combination of computational tools, in vitro analyses, and in vivo xenograft studies offers a vigorous frame for evaluating the potential of these drugs in a therapeutic context.

 The repurposing of clinically approved drugs as EGFR allosteric inhibitors provides a promising pathway to overcome drug resistance in NSCLC. This approach leverages known safety profiles, accelerating clinical translation. For this, the comprehensive methodology applied such as in silico, in vitro and in vivo is commendable. The molecular docking and dynamic studies are detailed and provide strong evidence of binding affinity for the EGFR allosteric site. Cytotoxicity, apoptosis, cell cycle arrest, and gene expression studies are systematically performed, showcasing the drugs’ mechanisms of action. The inclusion of tumor regression and histopathological analysis strengthens the translational relevance of the findings.

 The significant findings include both acenocoumarol and silodosin demonstrated effective inhibition of KRAS and ERK2, key players in EGFR signaling. The observed upregulation of caspase-3 and tumor growth inhibition validates the anti-NSCLC activity of these drugs. Further, the manuscript adheres to ethical guidelines, and all experimental procedures are well-documented, reflecting the integrity of the research.

 The findings in this work warrant publishing of this manuscript, however few suggestions for improvement are

 1) While the data are extensive, certain figures and tables lack concise labeling and clarity, which could hinder understanding for readers who are unfamiliar with the subject. 2) In the discussion part, readers could benefit from a more detailed comparison with existing EGFR inhibitors, emphasizing the advantages of acenocoumarol and silodosin in terms of efficacy, selectivity, and safety.

 3) Although the study concludes with a call for clinical trials, the discussion could elaborate on the specific challenges, such as pharmacokinetics and potential side effects, that need addressing for these drugs.

 4) Minor grammatical inconsistencies and redundancies could be refined to enhance the readability and professionalism of the manuscript.

Is the work clearly and accurately presented and does it cite the current literature?

Yes

If applicable, is the statistical analysis and its interpretation appropriate?

Yes

Are all the source data underlying the results available to ensure full reproducibility?

Yes

Is the study design appropriate and is the work technically sound?

Yes

Are the conclusions drawn adequately supported by the results?

Yes

Are sufficient details of methods and analysis provided to allow replication by others?

Yes

Reviewer Expertise:

Microbiology, Cancer Biology, Computational Biology

I confirm that I have read this submission and believe that I have an appropriate level of expertise to confirm that it is of an acceptable scientific standard, however I have significant reservations, as outlined above.

# Response

**Published:** 2025-09-15

**Authors:** Yogendra Nayak

**Affiliations:** Department of Pharmacology, Manipal Academy of Higher Education, Manipal, Karnataka, India

We thank the reviewer for the encouraging and insightful comments. The following revisions have been made based on the suggestions:

1. **Figures and Tables**: All figures have been relabeled for clarity. Figure legends are now more detailed, and axis labels/treatments are standardized.
2. **Comparison with existing EGFR inhibitors**: A paragraph comparing our findings with allosteric inhibitors like **EAI045** and **JBJ-09-063** has been added to the discussion.
3. **Discussion on clinical translation**: We now include a note on challenges related to pharmacokinetics, metabolism, and dosage limitations of repurposed drugs, particularly acenocoumarol (bleeding risk) and silodosin (hypotension).
4. **Language revision**: The manuscript has undergone thorough proofreading to remove redundancies and grammatical inconsistencies.

# Reviewer report: Reviewer response for version 1

**DOI:** 10.5256/f1000research.172916.r343035

**Published:** 2024-12-03

**Authors:** Tyler Beyett

**Affiliations:** 1 Department of Pharmacology and Chemical Biology, Emory University School of Medicine, Atlanta, USA

In this article, Maity et al. explore the effects of acenocoumarol and silodosin treatment on primarily on A549 cells. They show that high doses of either molecule induce apoptosis in cell culture and have a slight, but significant, effect in xenograft models. Overall, I have major concerns about the interpretation of the results. While I believe the data showing that these molecules are cytotoxic, I am not convinced that it is through inhibition of EGFR.

 Specific major points:

  

1. My biggest concern with this manuscript is the multiple references to these molecules as allosteric EGFR inhibitors. For example, in the manuscript title and the last sentence of the discussion. No data are presented in this study showing inhibition of EGFR by acenocoumarol or silodosin. There are also no data showing that they work through an allosteric mechanism of action. No allosteric inhibition data are provided in the reference prior work (reference 13) either. This reference only describes docking of the molecules and is lacking controls/reference molecules such as known allosteric EGFR inhibitors like EAI045 and JBJ-09-063. Docking is only a prediction that still requires experimental validation (protein-ligand binding analysis, biochemical kinase inhibition assay, cellular inhibition by phospho-EGFR western blot, etc.). Confirming an allosteric mechanism of action requires a crystal structure or enzymatic inhibition assays with varying ATP concentrations. For these reasons, the multiple claims that these molecules are allosteric EGFR inhibitors cannot be made, and this study must be presented/worded differently as a result.
2. Related to the previous point, since inhibition of EGFR has not been shown, it cannot be assumed that the cytotoxic effects observed are related to EGFR. Performing a target engagement assay would aid in showing the effects are on target rather than working through another mechanism/pathway to induce apoptosis. Many compounds will induce cell death.
3. Throughout the manuscript, critical details about experimental replicate (n) number are missing. The number of biological and technical replicates must be noted for all experiments. This is especially important since SEM is being reported, which is affected by n.
4. Throughout the manuscript, relevant controls including known EGFR inhibitors, ATP-competitive and allosteric, are missing. Cisplatin is not the standard of care for the vast majority of EGFR-driven cancers and thus is not an ideal reference compound. As A549 cells are do not express mutant EGFR (they overexpress wild-type EGFR), a pan-EGFR inhibitor like afatinib is an ideal positive control.
5. In the MTT cytotoxicity assay it is difficult to see where the IC50s are being derived from for some molecules. IC50 is defined as the inflection point of a sigmoidal curve, but many of these curves do not have such a point and do not appear to encompass enough of the dose-response curve to accurately report an IC50. Inclusion of EGFR inhibitors as controls, especially allosteric inhibitors, is needed for a frame of reference since IC50s are condition dependent. Additionally, it is not clear why acenocoumarol and silodosin were chosen. It is claimed that “From the cytotoxicity results, we selected the best drugs, acenocoumarol and silodosin, for further testing.” These molecules were not consistently more potent than the others. What wasn’t danusertib selected? It is most potent against the cancer cell line and least potent against the healthy cell line, which is typically the desired profile of a targeted therapy.
6. The section titled “KRAS and ERK2 enzymatic inhibition by acenocoumarol and silodosin” is misleading as no inhibition data are presented. That section only provides data on total protein levels. Furthermore, it appears as if this experiment was only performed once. Given the potential for bias in quantifying western blot bands by densitometry, especially weak bands, this experiment must be performed multiple times and error bars reported.
7. Related to the previous point, in the discussion it is stated that “Enzymes ERK2 and KRAS significantly inhibited enzymatic activity upon administration of silodosin” and “suppressing the signal transduction cascade.” This and related statements need to be removed, as enzymatic activity and signal transduction of ERK2 (typically assessed by phospho-ERK blotting) or activation of RAS (assaying for GTP bound state or GTPase activity) are not assessed. Furthermore, the use of “significantly” is inappropriate given that no statistical analyses were performed in the relevant figures 7 and 8.
8. Information on the statistical tests used in Figures 9, 10, and 11 are missing.
9. The related figures 7 and 8 do not make sense. For example, 12.5 uM silodosin greatly decreases expression, but the protein levels are not dramatically decreased. Similarly, for 25 uM acenocoumarol, expression is decreased for ERK2, but the protein level is normal. In the same group, KRAS expression is comparable to the controls, but protein levels are greatly increased. What is the explanation for this disconnect?
10. In figure 9, are statistical tests only performed for the last treatment point (day 21)? If so, this must be stated. Other time points are likely significant if also considered. In the figure legend, I hope that p<0.5 is a typo meant to be p<0.05. The former significance cutoff is not stringent enough.
11. In figure 11, what are the statistical significances for? And what statistical test was employed? Are you saying that all of the organs from a treatment group are significantly different than their corresponding values in the vehicle group? What about comparisons between treatment groups? Such analyses may require a multiple comparisons post hoc correction be used in the statistical analysis.
12. Numerous typos throughout including, but not limited to, T750M and L858M (page 3). There are also red underlines in the text for figure 14 that should be removed.
13. Antibodies used in western blots must be noted with their source and catalog number since they can vary widely.
14. Source data should include the individual replicates for each experiment in addition to just the mean ± SEM

Is the work clearly and accurately presented and does it cite the current literature?

Partly

If applicable, is the statistical analysis and its interpretation appropriate?

No

Are all the source data underlying the results available to ensure full reproducibility?

Partly

Is the study design appropriate and is the work technically sound?

No

Are the conclusions drawn adequately supported by the results?

No

Are sufficient details of methods and analysis provided to allow replication by others?

Partly

Reviewer Expertise:

EGFR pharmacology including allosteric inhibitor discovery and development

I confirm that I have read this submission and believe that I have an appropriate level of expertise to state that I do not consider it to be of an acceptable scientific standard, for reasons outlined above.

# Response

**Published:** 2025-09-15

**Authors:** Yogendra Nayak

**Affiliations:** Department of Pharmacology, Manipal Academy of Higher Education, Manipal, Karnataka, India

**Major Comment 1: Claim of allosteric EGFR inhibition without experimental validation.**

**Response: **We sincerely acknowledge this concern. The claim that acenocoumarol and silodosin are allosteric EGFR inhibitors was primarily based on in silico docking studies and prior literature suggesting possible interaction with the allosteric site. However, we recognize that docking studies alone do not suffice to label them as "allosteric inhibitors." We have accordingly:

- Revised the manuscript title, abstract, conclusion, and discussion to reflect a more evidence-aligned interpretation of the findings. The drugs are now presented as repurposed candidates with predicted interactions at EGFR-associated signaling levels, based on computational docking and preclinical evaluation. The title has been changed to: “ **Exploring acenocoumarol and silodosin in non-small cell lung cancer: Insights into EGFR-linked signaling mechanisms**”. This updated title avoids asserting mechanistic inhibition, emphasizes pathway-level relevance, and accurately reflects the scope and limitations of the study.
- Removed definitive language such as "allosteric inhibitor" and replaced it with "computationally predicted to bind to allosteric sites of EGFR."
- Cited the lack of direct evidence as a limitation and proposed further biochemical studies (e.g., kinase assays, phospho-EGFR western blots) in the future.

**Major Comment 2: No direct evidence that cytotoxicity is related to EGFR inhibition.**

**Response: **We agree. While we observed apoptosis and downregulation of KRAS and ERK2 components downstream of EGFR signaling, our current dataset does not establish a causal link between drug activity and EGFR inhibition. We have removed statements that imply a direct mechanistic link and instead suggest that the observed effects **may be associated** with EGFR signaling pathways, pending further validation. This clarification is incorporated into the discussion.

**Major Comment 3: Missing replicate (n) numbers and statistical clarity.**

**Response: **We apologize for the oversight. The manuscript has been revised to:

- Explicitly mention the number of **biological replicates (n=3)** for in vitro studies and **n=4 mice per group** for xenograft experiments.
- Clearly indicate **technical duplicates** where applicable.
- Ensure all SEM values are tied to biological replicates, not technical ones.

**Major Comment 4: Use of inappropriate controls in EGFR assays.**

**Response: **We acknowledge the point regarding the use of cisplatin instead of a known EGFR inhibitor. Our intention was to benchmark general anticancer activity rather than EGFR-specific inhibition, given the exploratory nature of this study. This rationale is now mentioned in the Methods and Limitations sections. We agree that future studies must incorporate controls like **afatinib or osimertinib** to provide a valid comparative frame.

**Major Comment 5: Concerns with IC50 calculations and molecule selection.**

**Response: **The IC50 values were calculated using nonlinear regression via GraphPad Prism. While sigmoidal fitting may not be ideal for all curves due to cell line-specific response variability, the curve fitting and R² values are considered.

 Regarding molecule selection, **danusertib** was excluded from further testing due to **known off-target cytotoxicity and poor pharmacokinetic properties** in pilot studies, though it showed high potency. This is now clarified in the Results section.

**Major Comment 6: Mislabeling of Western blot data as enzyme inhibition.**

**Response: **We regret the terminology used. The sections titled “enzymatic inhibition” now read **“protein expression levels”** via Western blot. No enzymatic activity assay was performed. All conclusions based on these blots have been modified accordingly to describe expression rather than inhibition.

**Major Comment 7: Inaccurate claims of signaling pathway inhibition.**

**Response: **We have removed all claims of direct inhibition of KRAS and ERK2 enzymatic activity or pathway signaling. All references to "significant inhibition" have been qualified to reflect **expression changes**, not enzyme activity or pathway suppression, and we note the need for **phospho-ERK or GTP-RAS** assays in future studies.

**Major Comment 8: Incomplete statistical test descriptions.**

**Response: **Statistical tests have now been specified as **ANOVA with Tukey’s post hoc test** or **unpaired t-test**, as appropriate. Significance values and p-values have been revised throughout the figure legends. The p<0.5 error has been corrected to **p<0.05**.

**Major Comment 9: Disconnect between mRNA and protein levels.**

**Response: **This is a valid observation. We have acknowledged in the discussion that **post-transcriptional or translational regulation** may cause such discrepancies, and that one-time point assessments without time-course validation may limit interpretability. These are now added to the limitations.

**Major Comment 10: Clarification in Figures 9–11 and statistical interpretations.**

**Response: **Clarifications have been added regarding Statistical tests in tumor volume measurements (Figure 9), Significance at **multiple time points** (but analyzed at day 21), The error in p-value notation has been corrected. Organ weight data (Figure 11) now includes the statistical method and indicates whether post hoc corrections were applied.

**Minor Comments (combined response):**

- All antibody sources and catalog numbers have now been added to the Methods section.
- Typos including T750M/L858M (corrected to T790M/L858R) have been fixed.
- All red underlines and formatting errors in figures have been corrected.
- Raw data files now include individual replicates and are available on Figshare [ https://doi.org/10.6084/m9.figshare.24587592.v5].
